# Preoperative Immunotherapy in the Multidisciplinary Management of Oral Cavity Cancer

**DOI:** 10.3389/fonc.2021.682075

**Published:** 2021-07-01

**Authors:** Ramez Philips, Chihun Han, Brian Swendseid, Joseph Curry, Athanassios Argiris, Adam Luginbuhl, Jennifer Johnson

**Affiliations:** ^1^ Department of Otolaryngology-Head and Neck Surgery, Thomas Jefferson University, Philadelphia, PA, United States; ^2^ Department of Medical Oncology, Thomas Jefferson University, Philadelphia, PA, United States

**Keywords:** immunotherapy, oral cavity squamous cell carcinoma (OCSCC), preoperative, multidisciplinary (care or team), multimodality, head and neck squamouscell carcinoma (HNSCC), induction, window of opportunity

## Abstract

Despite advances in multimodal treatment for oral cavity squamous cell carcinoma, recurrence rates remain high, providing an opportunity for new therapeutic modalities that may improve oncologic outcomes. Much recent attention has been paid to the molecular interactions between the tumor cells with the adjacent peritumoral microenvironment, in which immunosuppressive molecular changes create a landscape that promotes tumor progression. The rationale for the introduction of immunotherapy is to reverse the balance of these immune interactions in a way that utilizes the host immune system to attack tumor cells. In the preoperative setting, immunotherapy has the advantage of priming the unresected tumor and the associated native immune infiltration, supercharging the adaptive anti-tumor immune response. It also provides the basis for scientific discovery where the molecular profile of responders can be interrogated to elucidate prognostic markers to aid in future patient selection. Preoperative immunotherapy is not without limitations. The risk of surgical delay due to immune adverse events must be carefully discussed by members of a multidisciplinary treatment team and patient selection will be critical. One day, the discovery of predictive biomarkers may allow for algorithms where pre-surgical immunotherapy decreases the size of surgical defect and impacts the intensity of adjuvant therapy leading to improved patient survival and decreased morbidity. With further study, immunotherapy could become a key component of future treatment algorithm.

## Introduction

Oral cavity squamous cell carcinoma (OCSCC) affects approximately 34,000 people in the United Stated every year (1). The treatment of OCSCC often requires a multimodality approach involving surgery, radiation, and chemotherapy. The standard of care for locally advanced OCSCC is surgery followed by adjuvant radiation or chemoradiation based on adverse pathologic features of the primary tumor and involved cervical lymph nodes. Nonsurgical management is usually reserved for unresectable or inoperable tumors (2, 3). Therapeutic decisions incorporate patients’ oncological, functional, medical, and quality of life needs. Individualized treatment requires multidisciplinary management by a team that includes a head and neck surgeon, medical oncologist, radiation oncologist, speech therapist, nutritionist, and social worker to address patient-specific needs. Despite advancement in treatment modalities and multidisciplinary approaches, recurrence in advanced OCSCC remains elevated, with a 50-70% 5-year disease-free survival rate, providing an opportunity to incorporate novel therapeutic options that have potential to improve oncologic outcomes (4–7).

The value of immunotherapy in recurrent/metastatic head and neck squamous cell carcinoma (HNSCC) has been supported by multiple clinical trials (8). Immunotherapy enhances survival with fewer significant side-effects when compared to chemotherapy in this setting. Given these encouraging results in recurrent and metastatic disease, the role of immunotherapy is being evaluated in locally advanced OCSCC. Immunotherapy has particular appeal in the preoperative setting, as it can supercharge the native immune infiltrate within the peritumoral microenvironment, creating a powerful antitumoral response that can last beyond surgery and decrease the risk of local, regional, and metastatic recurrence. Although the introduction of preoperative immunotherapy in OCSCC management has exciting implications, it comes with its own challenges as we continue to understand its role in multidisciplinary treatment.

In this review, we discuss preoperative treatment in OCSCC, the rationale for using immunotherapy specifically in the preoperative setting, and the limitations of this treatment. We finally summarize clinical trials involving preoperative immunotherapy and discuss its potential role in multidisciplinary management.

## Preoperative Therapies in OCSCC

Preoperative therapeutic modalities in OCSCC include window of opportunity trials (WOTs), induction therapy and neoadjuvant therapy. WOTs in the preoperative setting are designed with the purpose of scientific discovery rather than pure therapeutic intent. In WOTs, a biopsy is first performed, followed by administration of an investigational drug during a window of time before surgery. Then, after surgical resection, the final pathologic specimen is compared to the biopsy tissue taken before the experimental drug was administered. This allows for assessment of the degree of tumor response, identification of pertinent prognostic biomarkers comparison of tumor and peritumoral microenvironmental characteristics between the pretreatment and posttreatment specimens. Oral cavity cancer is usually easily amenable to biopsy and provides a good model for WOT.

Preoperative treatment involves administering chemotherapy to shrink the tumor before definitive treatment with either surgery, radiation or chemoradiation. This technique has garnered interest in the management of OCSCC related to desire to control micrometastasis and the desire to convert borderline unresectable tumor to resectable tumors. Unfortunately, phase III randomized trials and meta-analyses evaluating use of induction chemotherapy followed by surgery versus upfront surgery have failed to show improvement in locoregional relapse, disease-free survival, and overall survival in OCSCC (9–13). A randomized trial compared the impact of induction chemotherapy with 3 cycles of cisplatin and fluorouracil followed by surgery (n=99) versus upfront surgery (n=99) in stage T2-T4, N0-N2, previously untreated patients (13). After a median follow up of 11.5 months, there was no difference in overall survival, locoregional relapse, and distant metastasis between the two groups (*p =* 0.340; *p* = 0.634; *p* = 0.153, respectively). Another phase 3 trial, published by Zhong et al. compared induction chemotherapy with 2 cycles of docetaxel, cisplatin, and fluorouracil (TPF) followed by surgery (n=128) versus upfront surgery (n=128) in patients with stage III/IVa locally advanced resectable OCSCC. Similarly, there was no significant difference in overall survival (*p* = 0.918) or disease-free survival (*p* = 0.897) between patients receiving induction chemotherapy versus upfront surgery. A meta-analysis of the previous two studies confirmed lack of difference in locoregional recurrence, overall, and disease-free survival between patients receiving induction chemotherapy versus upfront surgery (9). Further subgroup analysis of individual data from cN2 patients showed statistically significant overall survival benefit in favor of induction chemotherapy. Furthermore, studies have shown a high toxicity profile related to induction chemotherapy (11, 13). Rates of grade 3/4 adverse events in the above cited clinical trials ranged from 6.6 to 37% with 3 associated fatalities (3%) reported in the Bossi et al. clinical trial (11–13).

Induction chemotherapy’s toxicity profile combined with lack of demonstrable oncologic improvement has resulted in this strategy falling out of favor in the treatment of OCSCC. Nevertheless, the rationale and need for preoperative systemic treatment still holds and immunotherapy offers an enticing alternative for preoperative treatment.

## Immune Response in OCSCC

**Figure 1:**
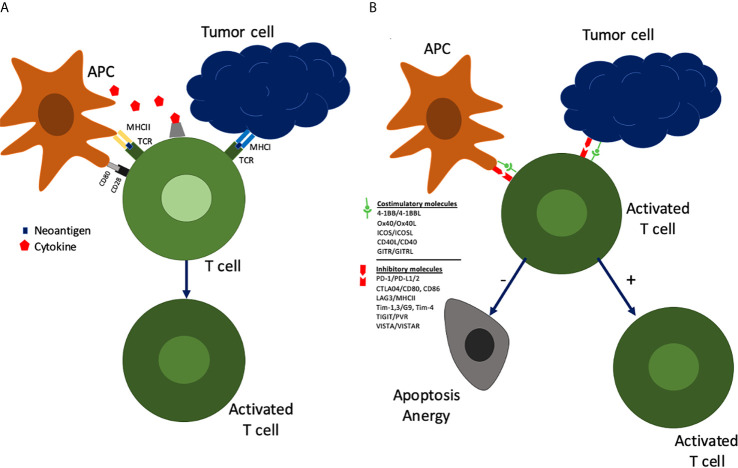
Tumor cells and antigen presenting cells (APCs) interact with immature T cells in a series of signals leading to their activation and subsequent modulation.

To understand the rationale for immunotherapy in HNSCC and more specifically OCSCC, an evaluation of the immune system and how it interacts with the tumor is essential. The initial tumor response is non-specific and is characterized by the innate immune system consisting of dendritic cells, macrophages, and natural killer cells. The adaptive immune response, characterized by lymphocytes, is subsequently activated by the presentation of tumor neoantigens *via* major histocompatibility complex (MHC) proteins to T cells. Tumor cells and antigen presenting cells (APCs) signal immature T cells leading to their activation and subsequent modulation (**Figure 1**). In the first signal the human leukocyte antigen (HLA) complex interacts with the T cell leading to their proliferation. A second signal *via* CD80/CD28 molecules leads to activation of T cells into tumor specific cytotoxic CD8+ T cells or helper CD4+ T cells. These activated T cells can further be modulated by costimulatory or inhibitory molecules. Costimulatory molecules can lead to maintenance of activation while inhibitory checkpoints lead to anergy/senescence or apoptosis of T cells. In concert, these signals create a nuanced response by the immune system to tumoral neoantigens.

HNSCCs evade their host immune response mechanisms through various molecular-level techniques (14). These techniques can be categorized into factors related to 1) the tumor (HLA mutations, cytokine release, checkpoint inhibition, costimulatory molecules) and 2) the tumor microenvironment (**Table 1**).

**Table 1:** Methods of immune evasion.

Tumor related factors		Tumor microenvironment	
MHC/APM mutations		Myeloid-derived suppressor cells (MDSCs)	
Immunosuppressive cytokine release (TGF-β, IL-6, IL-10)		Regulatory T cells (Tregs)	
Upregulating (checkpoint) inhibitor molecules		Tumor-associated macrophages (TAMs)	
Downregulating costimulatory molecules		Cancer-associated fibroblasts (CAFs)	

MHC, major histocompatibility complex; APM, antigen presenting machinery.

OCSCC has a high mutational burden related to the DNA damage caused by smoking and alcohol. Thus, tumor progression in the face of an intact immune system relies on natural selection of mutations that aid in immune system evasion. Particularly, mutations in the HLA and antigen processing machinery (APM) in OCSCC are important in escaping the immune system (15, 16). As described above, the HLA/APM complex is the first signal in activating T cells for eradication of tumor cells (17). Importantly, a *complete* loss of HLA/APM complex is a stimulator for NK cells to target and eradicate tumor cells (18).Therefore, to evade the immune system successfully, a mutation of the HLA/APM complex needs to alter protein structure and expression without causing complete loss of function. This alteration compromises the first signal of T cell activation and dampens the adaptive immune response aiding in tumor progression.

As highlighted above, inhibitory molecules can induce the adaptive immune response into a state of senescence. Immune checkpoint inhibitors play a role in tightly regulating immune activation to prevent autoimmunity and prolonged inflammatory states (15). Two prominent immune checkpoints receptors include programmed death 1 (PD-1) and cytotoxic T lymphocyte antigen-4 (CTLA-4). PD-1 is expressed in effector and regulatory T-cells and interacts with two ligands: PD-L1 and PD-L2. The interaction between PD-1 and PD-L1/2 induces T cell exhaustion, down regulation, and subsequent adaptive immune tolerance (15, 19). Therefore, high tumor expression of PD-L1 can lead to tumor evasion. In keeping with this theory, PD-L1 is expressed in up to 83% of OCSCC (15–17, 20, 21). Anti-PD1/PD-L1 agents boost the antitumor response by inhibiting the immunosuppressive signaling of these immune checkpoint signals (16). Pembrolizumab and nivolumab, IgG4 monoclonal antibodies that target PD-1, are currently approved for recurrent/metastatic HNSCC based on well documented efficacy in clinical trials (22, 23). The success of these compounds in recurrent/metastatic HNSCC provides a rationale for their introduction in preoperative treatment in OCSCC.

Similarly, CTLA-4 is expressed on T cells and interacts with the CD80/CD86 on antigen presenting cells (APCs) by competing with its stimulatory counterpart CD28 to primarily inhibit differentiation of naïve T-cells (24). CTLA-4 is upregulated in OCSCC and plays an important role in immune evasion (25). Inhibition of CTLA-4 can lead to infiltration of the tumor microenvironment with T cells and increases antitumor response (26). Multiple monoclonal antibodies against CTLA-4 have been developed to take advantage of this mechanism; ipilimumab was the first immune checkpoint inhibitor to be approved by the Food and Drug Administration (FDA) for the treatment of metastatic melanoma (27). Unlike PD-1 inhibitors, CTLA-4 inhibitors are not approved for use in HNSCC at this time, although multiple trials are currently underway. CTLA4-directed agents may have a role to play in the future for OCSCC based upon these results.

Other important novel immune checkpoints include lymphocyte activation gene-3 (LAG-3), T-cell immunoglobulin and mucin containing protein-3 (TIM-3), and B7 Homolog 3 (B7-H3) (28, 29). LAG-3 is expressed on exhausted T cells and interacts with tumor cells to inhibit cytotoxic T cell signaling. Inhibition of LAG-3 can lead to recruitment of cytotoxic CD8+ T cells and has shown to restrain tumor growth in *in vivo* models (28). Current clinical trials are investigating the role of anti-LAG-3 antibody (BMS-986016) with and without anti-PD1 in solid tumors including HNSCC (NCT01968109). Similarly, TIM-3 is expressed on lymphocytes and can lead to negative regulation of T helper cells and exhaustion of cytotoxic CD8+ T cells in HNSCC (29). *In vivo* models have shown that anti-TIM-3 therapy leads to tumor suppression. Anti-TIM-3 therapies are also being investigated in advanced solid tumors, but their specific role in HNSCC has not been elucidated. Finally, B7-H3 is one of the newest modulators of T-cell response and is expressed in head and neck cancer. This molecule has a co-stimulatory role and is thought to be utilized by cancers to evade immune activation (30). Phase 2 clinical trials are investigating the role of anti-B7-H3 molecules in HNSCC (NCT04634825).

Another tumor-related mechanism involves the suppression of immune costimulatory pathways, which would normally promote immune activation. A prominent molecule in the costimulatory pathway includes OX40, which is expressed on T cells and leads to their proliferation (15, 31). OX40 requires interaction with its ligand to induce antitumor immune activity. In HNSCC, the OX40 ligand is reduced on the tumor itself leading to suppression of immune activation, despite OX40 overexpression on host T cells (32, 33) (34). CD137, another costimulatory molecule, is expressed on cells of the adaptive immune system (T cells, NK cells) and promotes a healthy immune response (35). As such, agonistic CD137 therapy has shown to reduce tumor growth in a OCSCC mouse model (36). Additionally, the combination of costimulatory molecule agonists and check point inhibitor antagonists is being investigated in clinical trials (37).

Tumor cells have also evolved to produce cytokines to silence the adaptive immune response and lead to tumor cell progression. Particularly, OCSCC cells secrete inflammatory immune suppressive cytokines such as transforming growth factor (TGF)-β, interleukin (IL)-6, and IL-10 (38). These cytokines alter T cell signaling, subsequently suppressing their effector function (39–41). Immune activating cytokines are understandably reduced *via* tumoral mechanisms in OCSCC. A well-studied, downregulated cytokine is IL-2, which normally promotes innate and adaptive immune system activation. To address this deficiency, IRX-2, a homologous cell-derived complex multi-cytokine biologic preparation, consisting of active IL-2, IL-1β, gamma interferon (IFNγ), and tumor necrosis factor-α (TNF-α), is being studied in phase I/IIa trials in OCSCC and has shown promise (42–44). IRX-2 acts on T cells by preventing tumor-induced apoptosis and enhances its effector function, particularly in regional lymph nodes, to reinvigorate both innate and adaptive host immunity in the battle against tumor cells (44). A phase IIb trial (NCT02609386) in which 97 patients with stage II-IV OCSCC received perilymphatic injection of IRX-2 recently demonstrated a significant increase in immune response, particularly cytotoxic CD8+ T cells (45).. Its immune restorative effect on regional lymph nodes is particularly attractive in combination with other checkpoint inhibitors. The trial remains active and is pending final endpoint results on event-free and overall survival.

As highlighted above, tumor cells have evolved to alter their own immunogenicity through selected mutations and production of immunomodulatory mediators. These mechanisms occur in the context of a tumor microenvironment which contains a variety of immunomodulatory cells. Immunomodulatory cells create an immunosuppressed environment permissive of tumor progression. Such cells include regulatory T cells (Tregs), myeloid-derived suppressor cells (MDSCs), tumor-associated macrophages (TAMs), and cancer-associated fibroblasts (CAFs) (15). These cells express immunosuppressive cytokines, immune checkpoint inhibitors, and work in concert to create a microenvironment that silences the immune system and permits tumor growth (39, 46–53). Targeting of these immunomodulatory cells can alter the tumor microenvironment and promote an immune anti-tumor response. Tregs express previously discussed immunosuppressive cytokines such as TGF-β and IL-10, which function to undermine T cell function (39). Monoclonal antibodies against receptors on Tregs have been developed to deplete levels of Tregs in the tumor microenvironment; one example is Mogamulizab, a monoclonal antibody which targets CCR4 on Tregs, showed promising results in lung/esophageal cancer patients and is currently being investigated in HNSCC (54). Another method of targeting immunomodulatory cells, particularly MDSCs and TAMs, includes cell differentiation into an alternative phenotype. MDSCs are immature myeloid-derived cells that play a role in T cell suppression. Targeting MDSCs for the differentiation into their mature phenotype can inhibit their immunosuppressive nature (55). TAMs consist of M1 and M2 TAMs that represent opposing roles in immunomodulation. M2 TAMs are associated with oncogenic properties. In contrast, M1 TAMs are associated with tumor suppressive properties. The peritumoral microenvironment around OCSCC commonly shows an oncogenic increased ratio of M2: M1 TAMs, providing an opportunity to target M2 TAMs and alter the M2:M1 ratio towards a more tumor-suppressive phenotype (56, 57). Finally, an alternative strategy involves targeting chemotactic receptors in TAMs and MDSCs to prevent their recruitment in the tumor microenvironment and suppress tumor growth.

## Rationale for Preoperative Immunotherapy in OCSCC

As highlighted above, OCSCC has evolved molecular mechanisms to escape the host immune system and alter the peritumoral microenvironment to provide ripe conditions for tumor progression. These molecular changes provide the basis for exploring how immunotherapy might fit into the treatment algorithm for OCSCC. In the following section, we address the rationale for immunotherapy particularly in the preoperative setting.

### Clinical Advantages

**Figure 2:**
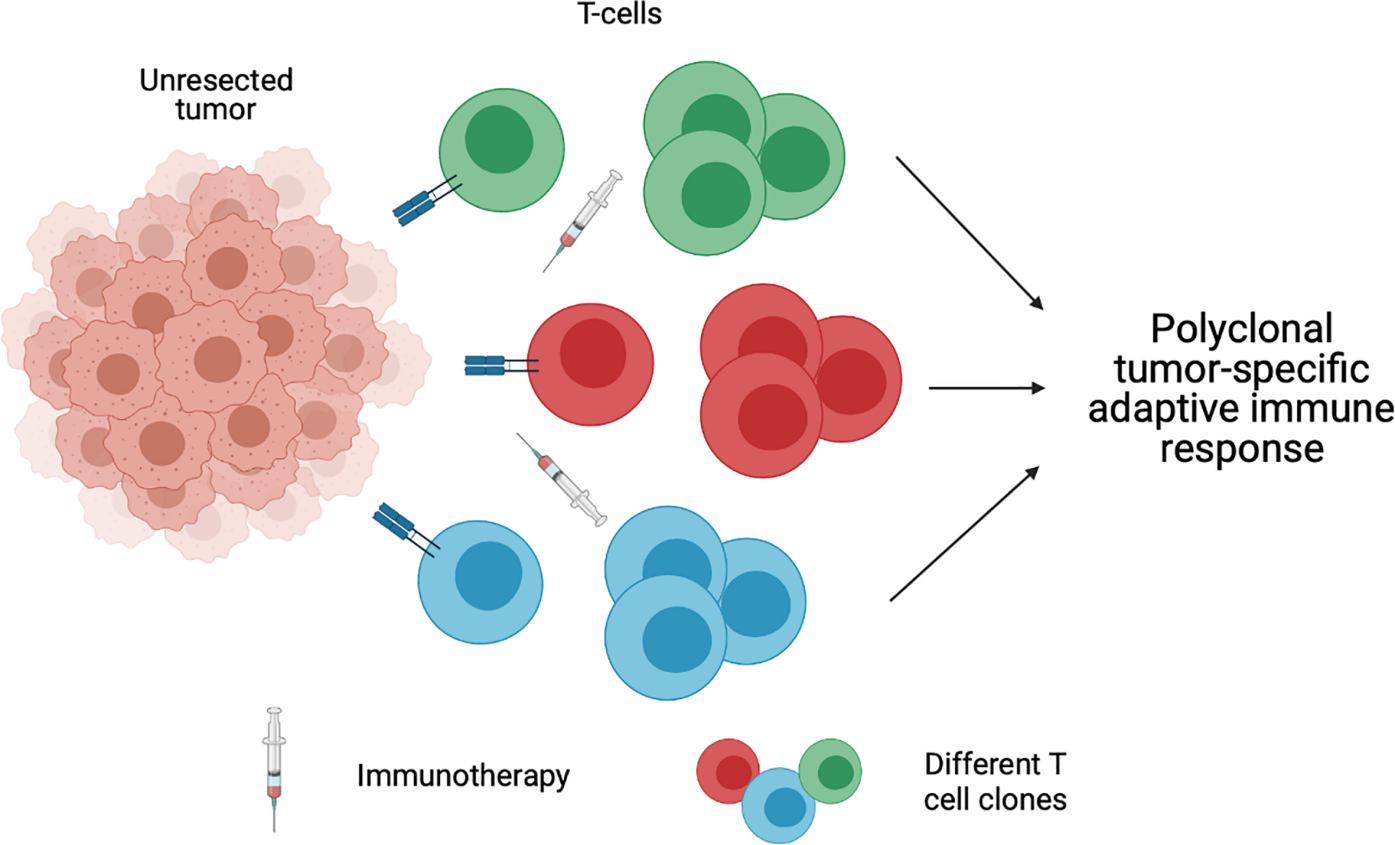
An unresected tumor bed provides diverse neoantigens, which leads to polyclonal activation of mature T cells and subsequent activation of a strong adaptive immune response. Created with BioRender.com.

The high recurrence rate in patients with OCSCC can be attributed to the presence of micrometastasis beyond the surgical field. Early systemic immunotherapy may enhance the eradication of micrometastatic deposits that lie outside of the planned surgical field, with potential to decrease rates of local, regional, or metastatic recurrence Although this advantage is present in any preoperative systemic therapy, preoperative immunotherapy has the advantage of a more tolerable side effect profile than that of traditional cytotoxic induction chemotherapy as discussed above. Patients who are treated with immune checkpoint inhibitors may therefore be more likely to tolerate subsequent aspects of their multidisciplinary care. The application of immunotherapy in the pre-operative setting rather than post-operatively or in the recurrent/metastatic setting also provides the distinct advantage of an intact tumor bed (**Figure 2**). As discussed above, the ability to effectively reinstate an adaptive immune response is based on presence of neoantigens and subsequent activation of T cells. In an unresected/unmanipulated tumor bed, there is an abundance of neoantigens (58). In the setting of preoperative immunotherapy, the presence of diverse numerous neoantigens can lead to polyclonal activation of mature T cells and subsequent activation of a strong adaptive immune response, which can have lasting effects beyond surgery (58, 59). Thus, the addition of preoperative immunotherapy to the current standard of treatment illustrates a method of treatment intensification in a cancer that has been proven difficult to cure. At the same time, however, for those patients who respond robustly to preoperative treatment, less morbid surgical approaches may also become an option.

### Biomarker Discovery

In addition to the clinical advantages listed above, pre-operative immunotherapy trials can provide a research mechanism for biomarker discovery. A phenomenon noted in the use of immunotherapy is the inter- and intra-tumor heterogeneity in response to immunotherapy (60). Clinical trials have shown that the range of pathologic response to immunotherapy is wide and correlates with recurrence rates between patients (61). Interestingly, studies have shown that even within the same patient, immunotherapy can have a different effect on the primary tumor bed and regional metastasis, highlighting the complexity of the tumor immune landscape and its implication on treatment response and prognosis (60, 62).

Providing preoperative immunotherapy in WOT allows for assessment of pathologic response. Profiles of responders can be compiled from the pretreatment specimens and interrogation of the posttreatment samples can allow for mechanistic and prognostic insights (63). Although WOT are not designed for justification to change standard of care, they can provide the pilot data for further trials and relatively short turn around on their correlative primary endpoints. The discovery of prognostic biologic markers can lead to individualized patient-centered treatment based on the patient’s unique immune profile. Currently, there are no validated tumor signature markers that can accurately predict response (64, 65). A list of tumor biomarkers being studied in preoperative immunotherapy clinical trials is listed in **Table 2**.

**Table 2:** Biomarkers assessed in clinical trials using preoperative immunotherapy in advanced oral cavity squamous cell carcinoma.

Trial number	Patient population	Preoperative immunotherapy	Biomarkers
NCT02296684 (61)	Stage III-IVb (AJCC, 7^th^ ed) HPV- HNSCC	Pembrolizumab	PD-L1 IFNγ pathway, immune and inflammatory genes (*IFNG, CXCL9, CXCL10, CXCL11*), T-cell checkpoint molecules, (*PDCD1, CTLA4, ICOS, TIGIT, IDO1*, and *TNFSF4)*, M1 macrophages CD4 and CD8 T cells
NCT02919683 (66)	≥T2-4b or N+, M0 (AJCC, 7^th^ ed) OCSCC	Nivolumab ± ipilimumab	CD4+ T cells
NCT03021993 (67, 68)	Stage II-IVA OCSCC	Nivolumab	CD26, Tim
NCT02641093 (69)	T3-4 and/or >2 + LNs HPV- HNSCC	Pembrolizumab	PD-1, PD-L1, CD8+ T cells
NCT02274155 (70)	Stage III-IVa HNSCC	Anti-Ox40 (MEDI6469)	CD4+ T cells; CD103+ CD39+ CD8+ T cells
NCT03003637 (71)	T3-4, N0-3, M0 HPV- HNSCC	Nivolumab ± ipilimumab	Endothelial cell and NK cell gene expression
NCT03129061 (72)	Locally advanced HNSCC	Nivolumab vs pembrolizumab	Novel radiofluorinated AraG imaging agent, [^18^F]F-AraG (Cellsight)

## Limitations of Preoperative Immunotherapy Treatment

Although the implications of immunotherapy in treatment of OCSCC are enticing, limitations should not be understated. While the side effect profile of immunotherapy is considered to be more tolerable than that of cytotoxic treatment, grade 3 and higher events have been reported in HNSCC (23, 42). Recently published trials have shown a low toxicity profile of monotherapy with immune checkpoint inhibitors in the preoperative setting, though grade 3/4 events can still occur, causing significant morbidity to individual patients (61).Trials highlighting the success of combination checkpoint immunotherapy or combination immunotherapy/chemotherapy have also established the increased toxicity related to targeting multiple pathways in the immune system (66, 73). Such side effects are sometimes subject to a variable length of treatment for full recovery (23, 74–76). Although very infrequent, adverse events can theoretically have implications on delay in surgical treatment and a detrimental effect on oncologic outcomes for individual patients (8). Besides the oncologic implications, complications can cause long-lasting comorbidities that require prolonged management beyond the period of cancer treatment (66). As such, preoperative immunotherapy must demonstrate oncologic benefit to justify the increase in patient morbidity in order to receive widespread acceptance.

In addition to the systemic side effects of immunotherapy, it is possible that immunotherapy could cause local treatment effect related to the recruitment of cells of the adaptive immune system and resultant debris. This can lead to issues with wound healing and subsequent surgical complications. A multi-institutional case series by Mays et al. aimed to elucidate the relationship between immunotherapy and surgical wound complications in patients undergoing ablative and flap reconstructive surgery. The results indicate an association between preoperative immunotherapy and major complications requiring invasive surgical treatment (OR 3.7; *p* = 0.048) and any type of treatment for complications in patients receiving preoperative immunotherapy (OR 2.9; *p* = 0.008) (77). Although there are inherent limitations in a retrospective case series with no true matched case-controls, the study findings highlight a need to assess the timing of immunotherapy in the preoperative period so as to limit potential surgical morbidity due to prior treatment.

The variability in patient response to treatment has become an important limitation to immunotherapy. The difference in tumor molecular landscape between patients provides the rationale behind the differences in treatment response. Recently, response to immunotherapy has been found to be discordant between primary tumor and lymph node metastasis in certain patients, illustrating the complexity of the immune landscape in OCSCC (62). A study by Merlino et al. compared treatment effect and radiographic volumetric response between primary tumor and lymph node metastasis within the same patient after receiving preoperative nivolumab prior to definitive surgery (62). This study identified a discordance rate of 50% between lymph node metastasis and primary tumor. Further analysis revealed that treatment discordance correlates with differences in local immune cell make up. This study highlights the challenges associated with a highly heterogenous disease.

Finally, a limitation of preoperative immunotherapy is the phenomenon of observed tumor growth noted by clinical or radiologic exam after initial treatment (78). When tumor enlargement is noted after immunotherapy, a distinction between true progression and pseudoprogression is required. True progression refers to an actual increase in tumor size after treatment. In contrast, pseudoprogression refers to a radiologic increase in tumor size after treatment and is related to inflammatory recruitment and necrosis as opposed to actual tumor growth (78, 79). In solid tumors, rates of pseudoprogression after immunotherapy is 10% (79). In particular, the rate of pseudoprogression is exceedingly rare in HNSCC (22, 23). KEYNOTE-012 and Checkmate-141, two trials studying pembrolizumab and nivolumab, respectively, in recurrent/metastatic HNSCC, reported 0.8% to 2.2% rate of pseudoprogression. There has been sparse data on rates of pseudoprogression after preoperative immunotherapy. An aggressive form of progression, termed hyperprogression, has also been identified and is characterized by an extremely rapid rate of tumor growth and is associated with poor prognosis (80). Similarly, hyperprogression is exceedingly rare after preoperative immunotherapy, with single digit cases reported in the literature. A phase II clinical trial (NCT03021993) studying preoperative nivolumab in OCSCC (NCT03021993) described only 1 out of 9 patient with hyperprogression, who ultimately received definitive surgery after the tumor doubled in size after immunotherapy treatment (67).

Unfortunately, the development of an accurate measure to distinguish pseudoprogression and true progression has proven to be difficult. Specific radiologic criteria have been developed to attempt to identify pseudoprogression (81, 82). The definition of hyperprogression has been variable in the literature and is based on a combination of time to treatment failure and tumor kinetics (80). Although rare in head and neck cancer, hyperprogression has detrimental implications to the patient. The etiology behind hyperprogression after immunotherapy has not been fully elucidated and debate whether it is a result of the natural history of an aggressive disease or poor response to immunotherapy is ongoing. In the preoperative setting, hyperprogression can have implications on the extent of surgical resection, subsequent treatment, treatment response, and likely survival outcomes in few patients. Thus far, there is no evidence that pseudoprogression or hyperprogression has led to delays in definitive treatment (8). In the context of a primary resectable OCSCC tumor, the rare occurrence of hyperprogression after immunotherapy can potentially make a resectable tumor unresectable, putting the patient at oncologic risk. This raises the issue of tolerance of complications in the salvage versus primary setting. In salvage treatment, the risk of oncological demise justifies certain complications from therapy. In the potentially curable primary OCSCC, such limitations might not be justified.

## Preoperative Immunotherapy 7WOT in OCSCC

With the rapid development of the field of preoperative immunotherapy, surrogates of early success in clinical trials are needed (58). There is a lack of consensus over which criteria best indicates positive tumor response (83). The difficulty with using imaging as a marker of tumor response is related to the phenomenon of pseudoprogression described above. After treatment, a radiographically detected mass can include both tumor cells and inflammatory cells/debris. Our recent publication correlating imaging with pathologic treatment effect in the context of a 4 week WOT supports the use of volumetric image analysis as it relates to evidence of pathologic response (62). The difficulty related to assessing tumor response has led to the advent of metabolic imaging studies (72). Such studies rely on the assumption that metabolic changes occur between radiographic changes. A recent clinical trial (NCT03129061) used a PET metabolic tracer ([^18^F]F-AraG), which preferentially accumulates in activated CD8^+^ T cells, to assess response to anti-PD-1 therapy (72). A patient with OCSCC had a 50% increase in [^18^F]F-AraG. This was seen with a concurrent increase in intratumoral CD4+ and CD8+ cells, illustrating the correlation between results of this new PET tracer and immune system activation. Additional clinical indicators of favorable outcome include overall and disease-free survival, which usually require large scale trials with long-term follow up. Therefore, pathologic complete response (pCR) and major pathologic response (MPR) were developed as markers of favorable outcome. pCR refers to the absence of tumor cells in the primary tumor site after surgical resection. Major pathologic response refers to the presence of <10% of tumor cells within the surgical specimen. Variations of pathologic response have also been used in clinical trials; pathologic tumor response (pTR) categorizes response into percentiles to include a wider range of response. There is a correlation of radiographic response to pathologic treatment response in head and neck cancer as early as 4 weeks of treatment (62). Recently, multiple clinical trials have reported favorable pathologic outcomes. A list of ongoing current trials, which include patients with OCSCC receiving neoadjuvant immunotherapy is presented in **Supplemental Table 1**. Initial results were published as conference abstracts in 2018-2019. As of 2020, multiple clinical trials have reported pathologic treatment response results in scientific manuscripts. Most clinical trials are not exclusive to OCSCC and include other HPV negative HNSCC. Two trials have published results in OCSCC exclusively and have reported favorable outcomes (66, 67). Clinical trials with reported results in a manuscript or a conference presentation are presented in **Table 3**. Here we discuss published scientific manuscripts studying preoperative immunotherapy in OCSCC.

**Table 3:** Results of published head and neck-specific clinical trials using preoperative immunotherapy.

Author	Trial Number	Phase	Patient population	OCSCC	Preoperative therapy	Pathologic and safety outcomes		
Uppaluri et al. (61)	NCT02296684	II	Stage III-IVb (AJCC, 7^th^ ed) HPV- HNSCC (n = 36)	61% (22/36)	Pembrolizumab (200mg) 13-22 days preoperatively	pTR 10%–49% = 22%		
						pTR ≥ 50% = 22%		
						MPR > 90% = 6%		
						Grade 3-4 AE = 0%		
Schoenfeld et al. (66)	NCT02919683	II	≥T2-4b or N+, M0 (AJCC, 7^th^ ed) OCSCC (n = 29)	100% (29/29)	Nivolumab (3mg/kg) wk 1 and 3 ± ipilimumab (1mg/kg) wk 1; Surgery wk 4	Nivo (n=14)		Nivo + ipi (n=15)
						pTR 10%–49% = 38%		pTR 10%–49% = 40%
						pTR ≥ 50% = 15%		pTR ≥ 50% = 33%
						MPR > 90% = 8%		MPR > 90% = 20%
						Grade 3-4 AE = 14%		Grade 3-4 AE = 33%
Zinner et al. (73)	NCT03342911	II	Stage III/IVA (AJCC, 8^th^ ed) HPV- HNSCC (n=26) Stage II/III (AJCC, 8^8h^ ed) HPV+ OPSCC (n=6)	81% (21/26)	Nivolumab (240mg) q 2 wks x 3	HPV- HNSCC:		
					Carboplatin q wk x 6	MPR > 90% = 65%		
					Paclitaxel (100mg/m^2^) q wk x 3	pCR = 42%		
						Grade 3-4 AE = 35%		
Horton et al. (67)	NCT03021993	II	Stage II-IVA OCSCC (n=9)	100% (9/9)	Nivolumab (3 mg/kg) q 2 weeks x 3/4	pTR > 30% = 44%		
						pCR = 0%		
						Grade 3-4 AE = 0%		
Wise-Draper et al. (69)	NCT02641093	II	T3-4 and/or >2 + LNs HPV- HNSCC (n=34)	Unknown	Pembrolizumab (200mg) x1 1-3 wks preoperatively	pTR ≥ 10% = 52%		
						pCR = 4%		
						Grade 3-4 AE = 3%		
Ferris et al. (84) (CHECKMATE-358)	NCT02488759	I, II	≥T1 and ≥N1	Unknown	Nivolumab (240mg) q2 weeks x2	No pathologic data		
			HPV+ (n=12)			Tumor reduction on CT scan = 48%		
			HPV- (n=17)			Tumor reduction 40 -75% = 13%		
			HNSCC			Grade 3-4 AE: HPV+ = 17%; HPV- = 12%		
Bell et al. (MEDI6469) (85)	NCT02274155	Ib	Stage III-IVA	Unknown	Anti OX40 antibody (MEDI6469) (0.4mg/kg) q2/3 days x3	No pathologic data		
			HPV+ (n=7)			Increased activation and proliferation of T cells		
			HPV- (n=11)			Grade 3-4 AE = 0%		
			HNSCC					
Zuur et al. (IMCISION) (71)	NCT03003637	Ib/II	T3-4, N0-3, M0 HPV- HNSCC (n = 12)	Unknown	Nivolumab (240mg) wk 1 and 3 ± ipilimumab (1mg/kg) wk 1	Nivo (n=6)	Niv + Ipi (n=6)	
						MPR >90% = 16.7%	MPR >90% = 33.3%	
					Surgery wk 5	pTR >50% = 0	pTR >50% = 16.7%	
						Grade 3-4 AE = 16.7%	Grade 3-4 AE = 33.3%	

AE, adverse events; AJCC, American Joint Committee on Cancer; HNSCC, head and neck squamous cell carcinoma; HPV, human papilloma virus; MPR; major pathologic response; OCSCC, oral cavity squamous cell carcinoma; pCR, pathologic complete response; pTR, pathologic tumor response.

### Preoperative Single Agent Immunotherapy

Most clinical trials in preoperative immunotherapy are investigating the use of single agent immunotherapy in OCSCC. Five of the 8 clinical trials with published results have focused on single agent immunotherapy. Of these 5 trials, 2 studied pembrolizumab, 2 studied nivolumab, and one studied the anti OX40 antibody, MEDI6469.

CHECKMATE 358 (NCT02488759) was the first clinical trial with published results presented in ESMO in 2017. Patients with ≥T1 and ≥N1 HNSCC were given 2 doses of nivolumab (240mg, IV every 2 weeks). Twenty-nine patients were enrolled, 17 of which had HPV negative tumors. Pathologic data has not been reported yet. Grade 3/4 adverse events were recorded in 2/17 patients with HPV negative tumors. Reduction of tumor noted by CT scan was seen in 6/13 of patients with HPV negative tumors. Since the presentation of CHECKMATE358, Wise-Draper et al. and Horton et al., have reported outcomes on preoperative single agents pembrolizumab, and nivolumab, respectively (67, 69). Favorable oncologic outcomes were reported with low rate of grade 3/4 adverse events. The success of these agents has led to clinical trials looking at different immunotherapies. Bell et al., published preliminary safety results on anti-OX40 antibody (MEDI6469) (85). There were no grade 3/4 adverse events reported.

More recently, Uppaluri et al. published another study focusing on use of neoadjuvant pembrolizumab (61). In this multicenter, phase II trial NCT02296684 (MK-3475-689), patients with surgically resectable stage III-IVb HPV negative head and neck cancer were randomly assigned to 1 of 2 arms (61). The first arm received 1 dose of pembrolizumab (200mg, IV) 13 to 22 days prior to definitive surgical resection. After surgery, patients received standard of care adjuvant treatment with the addition of pembrolizumab (200mg, IV, every 3 weeks for 6 doses) in patients with high-risk pathology (positive margins and extranodal extension). The second arm received 2 neoadjuvant doses (200mg, IV) 2 and 5 weeks preoperatively instead of 1 and did not receive any adjuvant pembrolizumab. The referenced manuscript highlights results from the first arm only. Primary endpoints included 1-year relapse rate in patients with high-risk pathology and pTR. Thirty-six patients, 61% of which had OCSCC, were enrolled in this trial. Pathologic response was observed in 44% of the patients (22% of pts with less than 10% pTR, 22% with 10-49% pTR). No patients had complete pathologic response after one dose, which can be related to monotherapy used in this trial, supporting the need for combination therapy. Predictive biomarker discovery will hopefully elucidate those that will benefit from monotherapy vs combinations. The one-year relapse rate amongst 18 patients with high-risk pathologic features was 16.7% (95% CI, 3.6% – 41.4%), lower than the historical average (35%). Relapse-free survival (RFS) in patients with >10% pTR was lower than patients with no pTR (61). This result supports the correlation between pathologic response and improved oncological outcomes. Importantly, use of pembrolizumab was not associated with any grade 3 or 4 adverse events or unexpected delay in surgery. Although this study showed the safety and lack of adverse events related to neoadjuvant pembrolizumab the small number of patients (n=36) makes drawing definitive conclusions difficult. Outcomes of the 2^nd^ arm in this clinical trial will be vital in assessing the most appropriate sequencing of immunotherapy (neoadjuvant versus adjuvant) for oncologic success. An international phase 3 trial is currently underway to evaluate the benefit of preoperative pembrolizumab in resectable, locally advanced head and neck cancer (NCT03765918).

### Preoperative Combination Immunotherapy

The favorable preliminary results seen in single agent preoperative immunotherapy has led to an increase in number of clinical trials with an interest in combination immunotherapy. Here we report the results of 2 clinical trials studying combination immunotherapy. The first trial studying preoperative combination immunotherapy was presented in ASCO in 2019 (71). The IMCISION trial (NCT03003637) was a phase 1b trial in T3-4, N0-3, M0 HPV negative HNSCC patients. Twelve patients were enrolled in this trial. Arm A received nivolumab (240mg IV) weeks 1 and 3. Arm B received nivolumab weeks 1 and 3 and ipilimumab (1mg/kg) week 1. Subsequently all patients received surgery week 5 followed by standard of care adjuvant therapy. In the 6 patients in Arm A, 1 patient had a near complete pathologic response (>90% response) and 1 patient had grade 3/4 colitis. In the 6 patients in Arm B, 3 patients had a pathologic response of >50%, 2 of whom had a near complete pathologic response (>90% response. Two patients had grade 3/4 adverse events, which included 1 case of colitis and 1 case of hepatitis.

NCT02919683 published in 2020 highlighted the value of combination therapy in preoperative OCSCC (66). In this phase 2 trial, OCSCC patients with T2-4b or node positive-disease with no distant metastasis received 2 cycles of nivolumab (3mg/kg at week 1 and 3 preoperatively) with or without ipilimumab (single dose at 1mg/kg at week 1 preoperatively). Patients then underwent surgery and received adjuvant therapy based on standard of care. The measured endpoints were safety/tolerability of the treatment and volumetric response (product of longest perpendicular bidirectional tumor measurements). Twenty-nine patients were enrolled (14 in nivolumab arm and 15 in nivolumab + ipilimumab arm). Volumetric response was observed in 50% (80% CI, 30.5% – 69.5%) of nivolumab only patients vs 53% (80% CI, 34.2% - 71.8%) in nivolumab + ipilimumab patients. Specifically, 38% of patients in nivolumab only arm vs 40% in nivolumab + ipilimumab arm had ≥ 10% and <50% pTR, while 15% of patients in nivolumab only arm vs 33% in nivolumab + ipilimumab had ≥ 50% pTR. Four patients had major/complete pathologic response (>90%.); one (8%) patient in the nivolumab alone arm and 3 (20%) patients in nivolumab + ipilimumab cohort. Seven patients had grade 3/4 adverse events including 2 grade 3/4 events in nivolumab only arm and 5 grade 3/4 events in nivolumab + ipilimumab arm.

Although the small sample size can be considered a limitation in the above studies, the rate of pathologic response in patients undergoing combination therapy is favorable compared to single agent immunotherapy as noted in the IMCISION trial and the above single agent trials. Nevertheless, as noted above, combination immunotherapy increases risk of adverse events. Therefore, although combination therapy can be effective, it comes at the risk of a worse side effect profile.

### Preoperative Immunotherapy and Chemotherapy

The value of combination therapy is not just limited to combination immunotherapy but also combined with chemotherapy and radiation. Out of the 8 currently published trials, only 1 looks at combination immunotherapy and chemotherapy. In a phase 2 trial (NCT03342911), patients with stage III-IVa HPV-negative head and neck cancer received preoperative nivolumab (240mg IV every 2 weeks for 3 doses), paclitaxel (100mg/m2 IV once weekly for 3 weeks), and carboplatin (IV weekly for 6 weeks) before definitive surgery (73). The primary endpoint was pCR at the primary site. Twenty-six patients, 81% of which were OCSCC, were enrolled in this study. This trial showed a favorable pathologic response with rate of pCR and MPR being 11/26 (42%) and 17/26 (65%), respectively. Importantly, this study highlights the effectiveness of combination immunotherapy with other well-established therapies (radiation therapy and chemotherapy) in the preoperative setting. Grade 3/4 adverse events were seen in 9/26 (35%) of patients but did not lead to delay of surgery. The higher toxicity rate in combining immunotherapy, radiation, and chemotherapy once again weighs against the oncological success of this therapy.

The promising results of preoperative immunotherapy have opened an avenue for an abundance in clinical trials. As highlighted above, combination checkpoint inhibitor therapy has favorable outcomes but can have higher rates of toxicity. This has led to the exploration of different methods of fighting the immunosuppressive environment in OCSCC. The introduction of intratumoral therapies, oncolytic viruses, and cancer vaccinations as tools to reinvigorate the immune system provides insight on the future of immunotherapy in OCSCC. This abundance of clinical trials aims to elucidate the molecular mechanisms of pathologic response and its relationship to long-term oncological outcomes including survival and recurrence. Although immunotherapy is an enticing new tool in multidisciplinary management of OCSCC, the field is still early in its development and will require in-depth scientific interrogation to solidify it as standard of care.

## Future Prospects

Clinical trials have largely focused on monoclonal antibodies for systemic delivery of immunotherapy in OCSCC. New approaches such as cancer vaccinations and adoptive cell transfer are under investigation (83).

Adoptive cell transfer includes harvesting T lymphocytes from an autologous source, expanding them with IL-2, identifying tumor-specific clones and reintroducing them to patients (86). Initial studies have shown success of adoptive cell transfer in metastatic melanoma refractory to chemotherapy. Patient response rate to adoptive cell transfer was reported at 56% in 93 patients with metastatic melanoma (87). Adoption of cell therapy is in its early stages in HNSCC primarily used in patients with recurrent/metastatic head and neck cancer. A phase II clinical trial evaluating adoptive cell therapy with autologous tumor infiltrating lymphocytes infusion followed by IL-2 for the treatment of patients with recurrent and/or metastatic squamous cell carcinoma of the head and neck is currently active (NCT03083873). A recent study by Ohtani et al., reported a 40% response rate in 5 patients with stage IV HNSCC after a mean follow-up of 26.2 months (88). Three patients received adoptive cell therapy after no response to conventional chemoradiation and 2 patients received therapy as adjuvant treatment. Although an enticing new technology, the road to adopting cell therapy in OCSCC, and specifically in the preoperative setting, is still theoretic in nature.

Vaccinations can induce an active immune response by presenting tumor antigens for T cell recognition and proliferation (86). Vaccines can be 1) whole-protein vaccines, which mimic tumor antigens and are subsequently presented to T cells, 2) whole-cell vaccines, which carry a higher antigen burden and consist of irradiated whole tumor cells, 3) autophagosome vaccines, which recycle cellular components and cross-present them to T cells and 4) oncolytic virus vaccines, which causes tumor cell lysis and subsequent presentation of tumor antigens to activate immune system (86). The accessibility of oral cavity tumors has made intratumoral delivery of vaccinations an attractive therapeutic option. Vaccinations have been particularly attractive in patients with HPV-related oropharyngeal squamous cell carcinoma due to druggable oncoproteins E6 and E7. Multiple clinical trials investigating the role of vaccines such as ISA-101 (targets E6 and E7) and ADX 11-001 (targets E7) are underway in HPV-related OPSCC (NCT02426892 and NCT02002182) with positive preliminary results (89). The introduction of vaccines in the OCSCC has lagged behind HPV-related oropharyngeal cancer. A window of opportunity trial (NCT04247282) is currently investigating the role of neoadjuvant TriAd vaccine along with anti-PD-L1/TGF-beta Trap (M7824) and anti-IL15 in patients with locally advanced, resectable stage II-IV HPV negative HNSCC. The TriAd vaccine consists of three adenoviral vaccines, ETBX-051, ETBX-061, ETBX-011, which target the 3 proteins, brachyury, mucin-1, and carcinoembryogenic proteins respectively. The primary endpoint is pathologic complete response after definitive surgery. The results of this study may prove important for the introduction of vaccinations in the preoperative setting of OCSCC.

## Integrating Immunotherapy Into Multidisciplinary Management

As highlighted in the above sections, the complexity of head and neck cancer starts at the molecular level and translates to the clinical level. This provides rationale behind the need for multidisciplinary management to provide optimal, patient-centered, individualized treatment that caters to patients’ specific needs. Multidisciplinary management of head and neck cancer care has been shown to improve outcomes in head and neck cancer (90). This is likely due to the wide array of specialists bringing diverse knowledge and skills, improvement in coordination and quality of care, decreased time to treatment, and important focus on cancer survivorship (90). As such, the National Comprehensive Cancer Network (NCCN), recommends that patients be treated at high-volume centers with multidisciplinary management and expertise (91).

The reinvigoration of immunotherapy has provided a promising addition to the armamentarium in the fight against OCSCC while also adding another layer to its complexity. It is important to acknowledge that immunotherapy is a developing field, and a significant amount of information is yet to be understood. Therefore, ongoing education with the latest research is key as the field is actively changing each day in order to provide the most up-to-date evidence-based-care to patients. Providers must be prepared to answer questions from the patients who may have access to the latest research. On the other hand, many patients may not be aware of these recent breakthroughs in immunotherapy and opportunities to participate in clinical trials allowing providers the opportunity to participate in patient education.

As knowledge surrounding immunotherapy increases, existing members of the multidisciplinary team will need to tailor their care to include immunotherapy-specific issues. As we continue to learn about the side effect profile of immunotherapy, a constant route of communication with the patient is essential in order to recognize these events early to treat and prevent any long-term harm to the patient. When these new adverse events do arise, there should be prompt recognition and intervention that may involve experts across different organ systems to minimize poor outcomes. In patients undergoing preoperative immunotherapy, coordination of care becomes of the utmost importance to ensure the correct timing of immunotherapy administration as it relates to surgery.

## Conclusions

**Figure 3:**
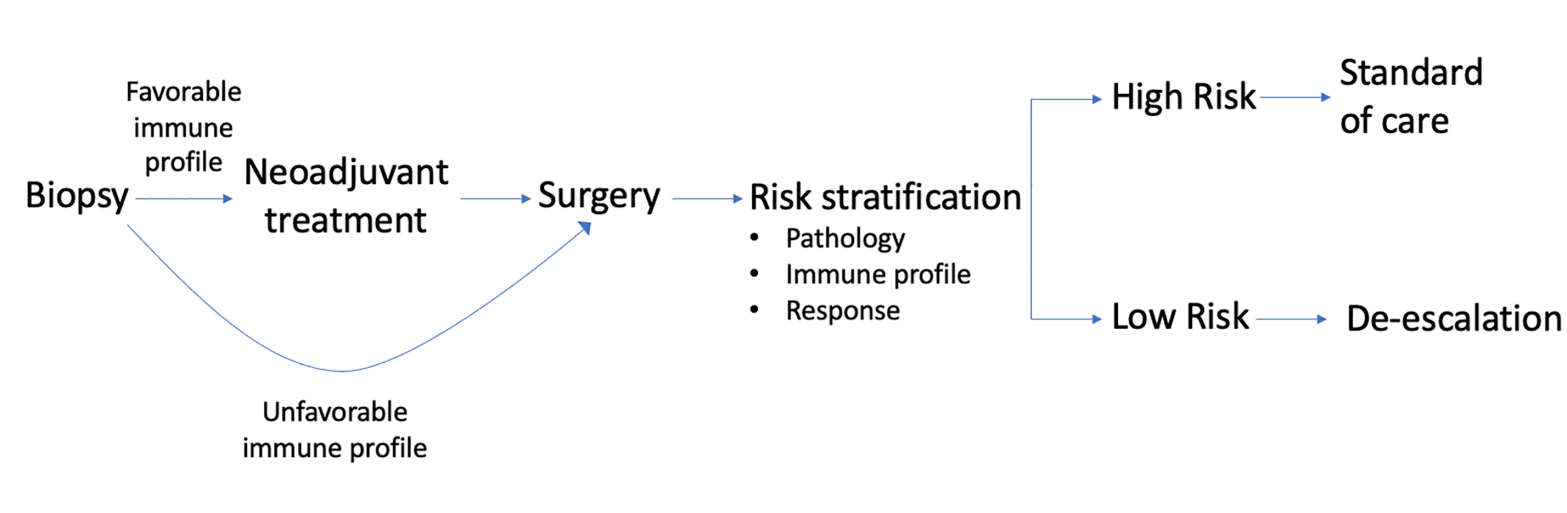
Immune profile of biopsied tumor can indicate need for preoperative immunotherapy in OCSCC. After definitive surgery, risk stratification can be done using pathologic data, immune profile, and response to initial treatment. Level of risk can then dictate adjuvant therapy.

Current clinical trials are investigating a novel role for immunotherapy as a method of treatment optimization in OCSCC. Ultimately, the field strives to improve oncologic, safety, and functional outcomes in these patients. Preoperative immunotherapy could provide an avenue both for treatment intensification (addition of systemic agents) and de-intensification (alteration of surgical approaches and subsequent adjuvant therapy. At this time, these advantages are currently hypothetical in nature. To truly achieve the goal of integrating immunotherapy into the standard treatment paradigm, patient selection becomes paramount. Current standards of treatment are deeply rooted in TNM staging. WOT clinical trials in OCSCC strive to identify predictive biomarker for patient selection and identification of targets for drug development (8). In the future we may have the ability to risk stratify patients based on multiple factors including pathology, response to preoperative immunotherapy, and immune profile to direct treatment (**Figure 3**). By generated individualized, tumor-specific algorithms to achieve better oncologic and functional outcomes we will one day be able to change the treatment of OCSCC for the better.

## Author Contributions

RP: Literature review, analysis, and interpretation, manuscript preparation, revision, and approval. CH: Literature review, analysis, and interpretation, manuscript preparation, revision, and approval. BS: Literature review, analysis, and interpretation, manuscript preparation, revision, and approval. JC: Literature interpretation, manuscript revision, and approval. AA: Literature interpretation, manuscript revision, and approval. AL: Literature interpretation, manuscript revision, and approval JJ: Literature interpretation, manuscript revision, and approval. All authors contributed to the article and approved the submitted version.

## Conflict of Interest

The authors declare that the research was conducted in the absence of any commercial or financial relationships that could be construed as a potential conflict of interest.

## Supplementary Material

Supplementary Table 1 Head and neck-specific clinical trials using preoperative immunotherapy in advanced oral cavity squamous cell carcinoma. AE, adverse effects; DFS, disease-free survival; DLT, dose limiting toxicities; EFS, event-free survival; LRR, locoregional relapse rate; ORR, objective response rate; MPR, major pathologic response; MRD, maximum tolerated dose; MTE, major treatment effect; PCR, pathologic complete response; PTR, pathologic tumor response; SBRT, stereotactic body radiation therapy; STN, significant tumor necrosis; VRR, volumetric response rate.
