# Supplementary material for: Preoperative Immunotherapy in the Multidisciplinary Management of Oral Cavity Cancer
Source: Front Oncol. 2021 Jul 1;11:682075. doi: 10.3389/fonc.2021.682075 (PMC8281120; doi:10.3389/fonc.2021.682075)
Supplement: Supplementary Table 1 — Head and neck-specific clinical trials using preoperative immunotherapy in advanced oral cavity squamous cell carcinoma. AE, adverse effects; DFS, disease-free survival; DLT, dose limiting toxicities; EFS, event-free survival; LRR, locoregional relapse rate; ORR, objective response rate; MPR, major pathologic response; MRD, maximum tolerated dose; MTE, major treatment effect; PCR, pathologic complete response; PTR, pathologic tumor response; SBRT, stereotactic body radiation therapy; STN, significant tumor necrosis; VRR, volumetric response rate. [file Table_1.docx]

**Supplemental Table 1.** Head and neck-specific clinical trials using preoperative immunotherapy in advanced oral cavity squamous cell carcinoma

| **Trial Number** | **Phase** | **Patient population** | **Preoperative therapy** | **Primary endpoint** |  |
| --- | --- | --- | --- | --- | --- |
| NCT03700905 | | III | Stage III-IVb HPV- HNSCC | Nivolumab | DFS |
| NCT03765918 | | III | Stage III HPV+ OPSCC, Stage III/IVa HPV- HNSCC | Pembrolizumab | MPR, EFS |
| NCT02296684 | | II | Stage III/ IV HPV- HNSCC | Pembrolizumab | LRR, distant failure rate, MPR |
| NCT02609386 | | II | Stage II-IVa OCSCC | IRX-2, cyclophosphamide, indomethacin, zinc-containing multivitamin, omeprazole | EFS |
| NCT02641093 | | II | T4 (any N) or ≥  N2 (any T) HPV-HNSCC; T3 (any N) OCSCC | Pembrolizumab | AE, DFS |
| NCT03003637 | | I/II | T3-4N0-3M0 HNSCC | Nivolumab +/- Ipilimumab | Surgery delay; PTR; impact of hypoxia on T-cell abundance |
| NCT03021993 | | II | T2-4 OCSCC, rT2-4 previously treated with surgery | Nivolumab | ORR |
| NCT03174275 | | II | Stage III/IV HPV+ & - HNSCC | Carboplatin, Paclitaxel, Durvalumab | PCR |
| NCT03247712 | | I/II | Surgically resectable HPV+ & - HNSCC | Nivolumab + RT | Surgery delay |
| NCT02827838 | | II | Surgically resectable  Stage I-IV OCSCC, OPSCC | Durvalumab | Systemic immune effects |
| NCT02997332 | | I | OCSCC | Durvalumab, Docetaxel, Cisplatin, 5-FU | Recommended phase 2 dose, number of dose limiting toxicity |
| NCT02882308 | | II | Surgically resectable HNSCC | Olarparib +/- Cisplatin +/- Durvalumab | Ki-67 change |
| NCT03342911 | | II | Stage III-IVa HPV- HNSCC; Stage II-III HPV+ HNSCC | Carboplatin, Paclitaxel, Nivolumab | PCR |
| NCT03708224 | | II | Stage III-IV HPV- HNSCC | Atezolizumab +/− Tiragolumab +/− Tocilizumab | CD+ T-cell infiltration; R0 resection |
| NCT03721757 | | II | T1-3N1-3 or T3-4N0 OCSCC | Nivolumab | DFS |
| NCT03737968 | | II | Stage II-IVb HNSCC | Durvalumab +/− Tremelimumab | LRR, distant metastatic rate |
| NCT03635164 | | I | Stage II-IV HPV- HNSCC (including stage II OCSCC) | Durvalumab + SBRT | MTD; DLT |
| NCT03843515 | | I | Stage III/IV OCSCC | Nivolumab | AE; SUV values on PET |
| NCT04247282 | | I, II | Stage II-IV HPV-HNSCC | Anti-PD-L1/TGF- β Trap (M7824) +/- TriAd Vaccine +/- anti-IL-15 (N-803) | PCR |
| NCT02919683 | | II | ≥ T2 or N+ OCSCC | Nivolumab +/- Ipilimumab | VRR |
| NCT02812524 | | I | Surgically resectable HNSCC | Ipilimumab | Surgery delay |
| NCT03765918 | | III | Stage III-IVa HPV- HNSCC; Stage III HPV+ HNSCC | Pembrolizumab | MPR; EFS |
| NCT02274155 | | I | Stage III-IVa HNSCC | Anti-OX40 (MEDI6469) | AE |
| NCT03916627 | | II | HNSCC | Cemipilimab +/- platinum doublet | MPR; STN; MTE |
| NCT03129061 | | I | Locally advanced HNSCC | Nivolumab vs Pembrolizumab | T cell activation |
| NCT02124850 | | I | Stage II-IVa HNSCC | Metolimod + Cetuximab +/- Nivolumab | Immune biomarkers |
| NCT04080804 | | I | Stage III-IVa HNSCC | Nivolumab +/- Ipilimumab +/- Relatlimab | AE |

Abbreviations: AE, adverse effects; DFS, disease-free survival; DLT, dose limiting toxicities; EFS, event-free survival; LRR, locoregional relapse rate; ORR, objective response rate; MPR, major pathologic response; MRD, maximum tolerated dose; MTE, major treatment effect; PCR, pathologic complete response; PTR, pathologic tumor response; SBRT, stereotactic body radiation therapy; STN, significant tumor necrosis; VRR, volumetric response rate
